# Delayed Diagnosis of Shoulder Septic Arthritis and Osteomyelitis During the COVID-19 Pandemic: The Role of Point-of-Care Ultrasound

**DOI:** 10.7759/cureus.35460

**Published:** 2023-02-25

**Authors:** Lachlan Driver, Kathleen McFadden, Nour Al Jalbout, Hamid Shokoohi

**Affiliations:** 1 Department of Emergency Medicine, Massachusetts General Hospital, Harvard Medical School, Boston, USA; 2 Department of Internal Medicine, Massachusetts General Hospital, Harvard Medical School, Boston, USA

**Keywords:** osteomyelitis, septic shoulder, covid-19, point-of-care ultrasound, septic arthritis

## Abstract

The diagnosis and treatment of septic arthritis are complex and require collaboration among multiple medical services, especially in the emergency department (ED). This case report highlights the difficulties in diagnosing shoulder septic arthritis, which is a rare condition in adults and can present with subtle symptoms. The patient was eventually diagnosed with septic arthritis of the left shoulder. However, the diagnosis was delayed due to the impact of the COVID-19 pandemic on obtaining an outpatient MRI and a previous shoulder injury that added confusion. Delays in diagnosis and treatment can lead to a rapid destruction of the affected joint, causing significant morbidity and mortality. This case report also highlights the importance of alternative diagnostic tools, such as point-of-care ultrasound (POCUS), which is quick, inexpensive, and may lead to earlier detection of joint effusions and prompt arthrocentesis.

## Introduction

The COVID-19 pandemic has brought numerous unheralded challenges to the medical field. One of these challenges is the significant delay in non-urgent outpatient imaging, with some hospitals reporting a 72% decrease in studies [1]. This may be compounded by physician challenges in determining which imaging needs to be obtained based solely on a virtual visit, as well as a patient's hesitation to present to the hospital due to a fear of acquiring COVID-19 or other infections [2]. Thus, in the setting of the COVID-19 pandemic, when a patient’s condition is considered non-urgent during a visit, imaging and further diagnostics could take months to obtain.

As patients are increasingly unable to see their outpatient providers due to clinic closures and scheduling delays, emergency physicians must be prepared to diagnose and treat these patients promptly. However, many emergency departments (EDs) in the United States are also operating at critical volumes [3]. In this setting, the ability to obtain rapid, accurate diagnostics is of even higher utility than in the past. Thus, the potential importance of point-of-care ultrasound (POCUS) for the diagnosis of time-sensitive pathologies, such as septic arthritis, is presented in this case.

## Case presentation

A 59-year-old man with a left rotator cuff repair in 2011 and non-insulin-dependent type 2 diabetes presented to the ED in June 2020 with a complicated 12-week history of subacute, progressive pain to his left shoulder following an exercise-induced injury. The patient attended a virtual clinic visit after four weeks of pain and obtained the recommendation to reduce his exercise program and to try non-steroidal anti-inflammatory drugs as well as acetaminophen. Citing the COVID-19 pandemic, he had difficulty seeing a healthcare provider in person. He attended virtual physical therapy appointments but was unable to achieve complete symptomatic relief.

After many weeks of scheduling obstacles, he underwent an outpatient MRI, which showed marked bone marrow edema adjacent to suture anchors in the greater tuberosity of the humerus as well as in the lateral acromion and posterior superior glenoid. There was significant intramuscular edema in the rotator cuff and deltoid with a small, loculated effusion along the greater tuberosity which extended beyond the suture anchors.

The patient was notified to present to the ED for further evaluation. At the time of ED presentation, the patient endorsed fatigue and chills, without fevers. He continued to endorse significant left arm pain and limitation in range of motion. An extensive infectious review of systems was otherwise negative.

The patient’s past medical history was notable for obesity, non-insulin-dependent type 2 diabetes, asthma, gastroesophageal reflux disease, hyperlipidemia, and hypertension. He reported a history of a left rotator cuff repair in 2011, for which orthopedic surgery used stainless steel anchors.

Vitals showed blood pressure of 121/82 mmHg, heart rate of 102 beats per minute, and respiratory rate of 18 breaths per minute. His temperature was 36.4°C and oxygen saturation was 98% on room air. On examination, the patient had severe left shoulder tenderness to palpation with a limited range of motion in external and internal rotation. He was unable to abduct or flex the left shoulder beyond sixty degrees but was otherwise neurovascularly intact. There was no overt edema or overlying erythema or demarcation overlying the affected shoulder. He was noted to have a normal range of motion in the right upper extremity.

**Figure 1:**
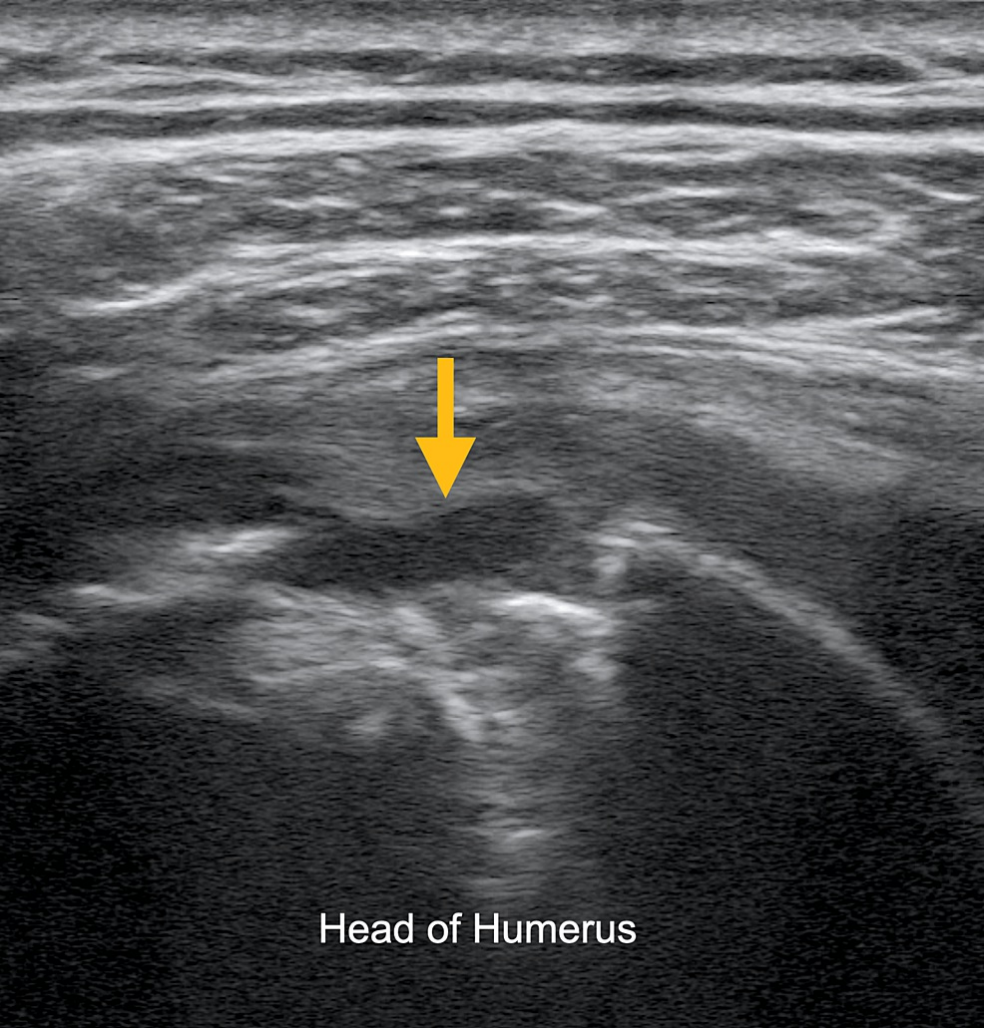
Ultrasound image of the left shoulder using a high-frequency linear probe revealed cortical erosion of the humeral head (arrow).

**Figure 2:**
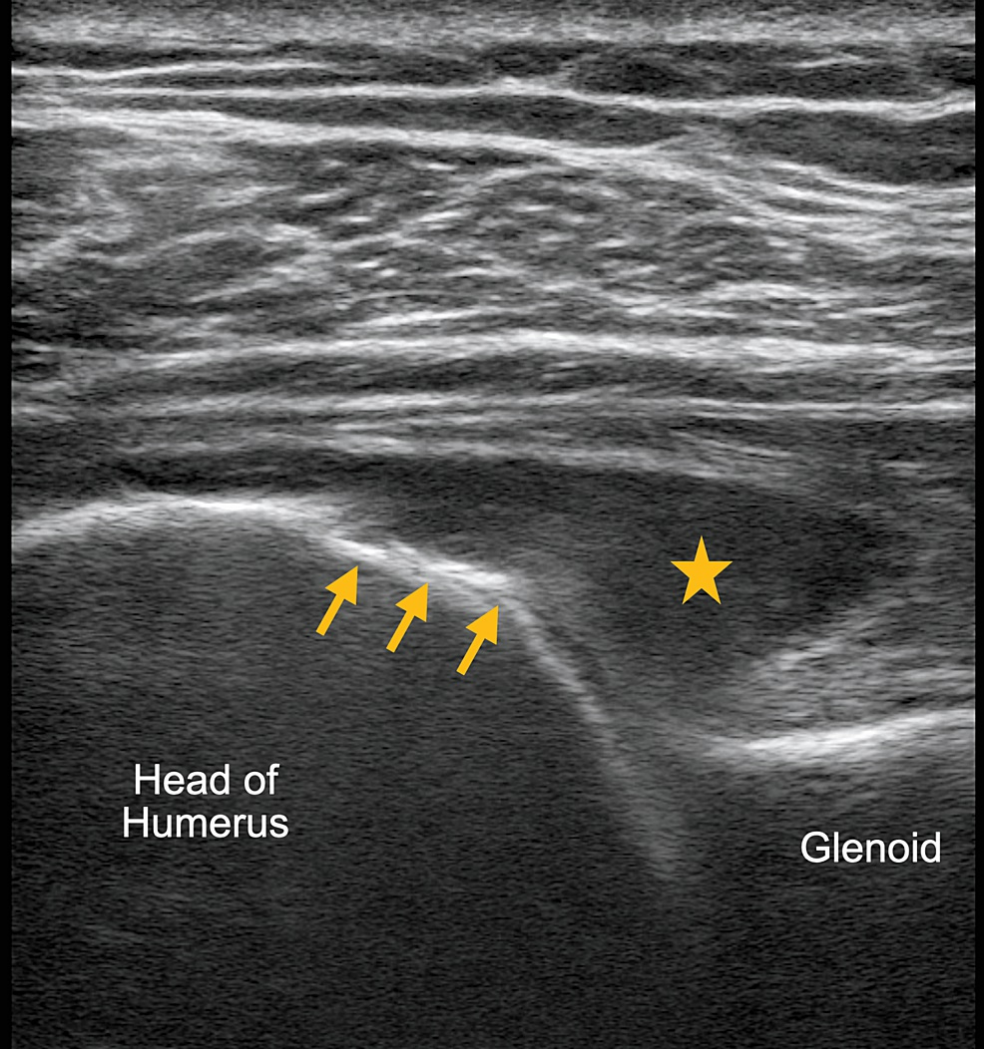
Ultrasound image of the left shoulder using a high-frequency linear probe revealed cortical erosion of the humeral head (arrows) with a joint effusion (star).

**Figure 3:**
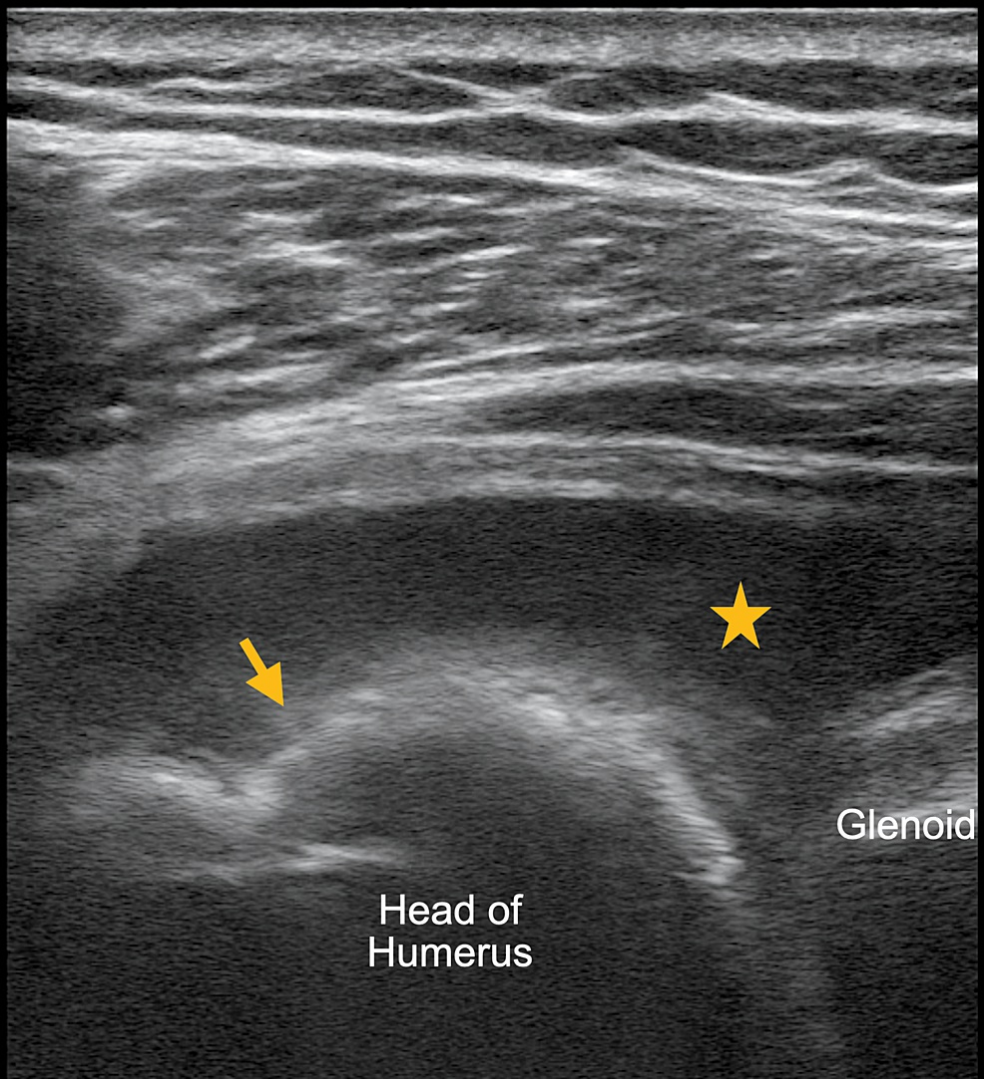
Ultrasound image of the left shoulder using a high-frequency linear probe revealed cortical erosion of the humeral head (arrow) with a joint effusion (star).

Laboratory data were notable for anemia with hemoglobin of 9.1 g/dl, white blood cell count of 8.1 K/µl with neutrophil percentage of 79.6%, and an international normalized ratio (INR) of 1.2. Though the patient reported he was sent in for concern about septic arthritis due to concerning MRI and interventional radiology (IR) biopsy results, the emergency physician did not have access to these data. Therefore, a POCUS was performed, which showed a left shoulder joint effusion with supraspinatus tendon injury and cortical erosion of the humeral head with periarticular edema as seen in Figures 1-3. 

 

 

**Figure 4:**
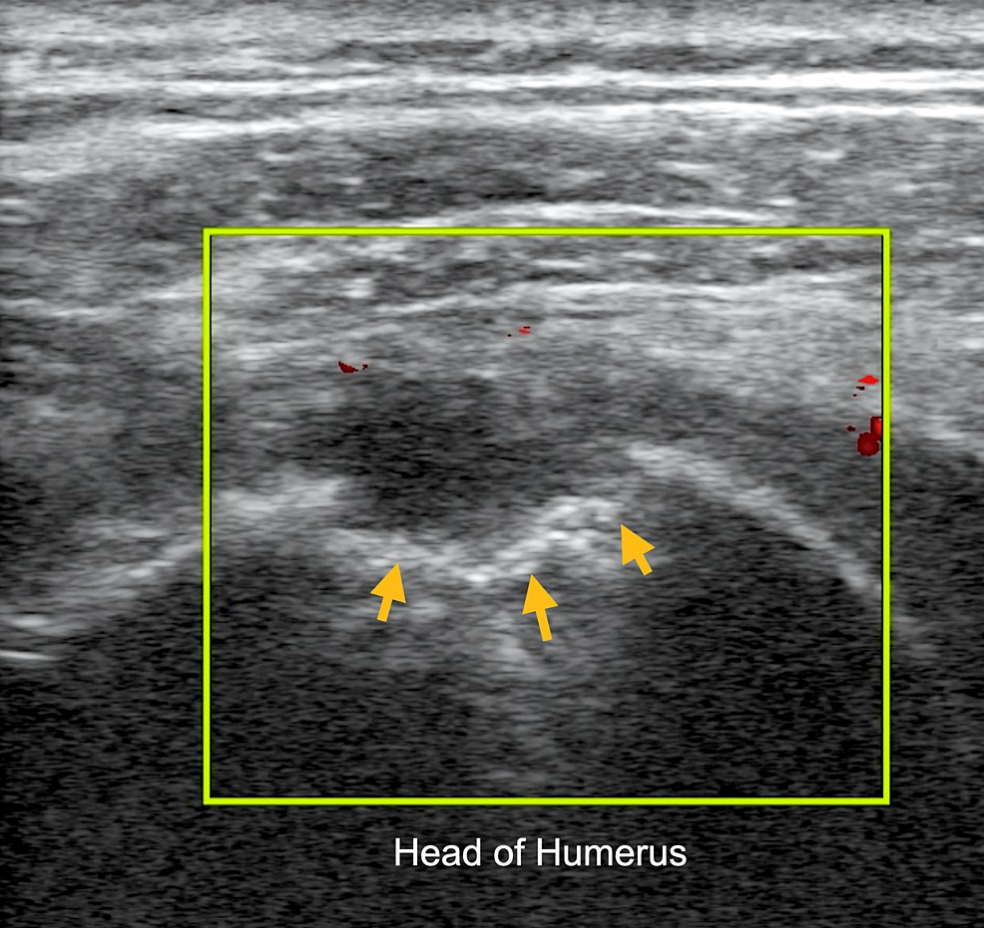
Ultrasound image of the left shoulder using a high-frequency linear probe with power Doppler applied revealed no flow around the cortical erosion of the humeral head (arrows).

Additionally, the ultrasound image of the left shoulder using a high-frequency linear probe with power Doppler applied revealed no flow around the cortical erosion of the humeral head as seen in Figure 4. Given these POCUS findings, the ED team consulted the orthopedic surgery team who recommended arthroscopic irrigation, which the patient underwent in the operating room (OR) the day after his ED presentation. During the procedure, both the glenohumeral and subacromial spaces were sampled under fluoroscopic guidance. Subsequently, cultures from the subacromial space grew Gram-positive cocci which were identified as being methicillin-sensitive *Staphylococcus aureus* (MSSA).

Subsequently, the patient was admitted to the orthopedic surgery service for left shoulder arthroscopic incision and drainage of the glenohumeral joint, subacromial space, and debridement of the humeral head, which he underwent without complication. He remained stable postoperatively and worked with physical therapy to increase upper extremity range of motion and strength thereafter. Antibiotics initially included vancomycin and cefazolin. Once cultures demonstrated MSSA, the patient continued cefazolin administration via a peripherally inserted central catheter (PICC).

## Discussion

Septic arthritis is a medical emergency associated with considerable morbidity because of the potential for rapid bone and cartilage destruction. The shoulder joint accounts for only 5-12% of all cases of septic arthritis [4]. However, it is associated with poor prognosis and frequent sequelae, including recurrent effusion and osteomyelitis. The standard treatment of septic arthritis includes early drainage of the glenohumeral joint and intravenous antibiotics, as prompt diagnosis and treatment are critical in achieving satisfactory outcomes [4]. Although MRI is the preferred imaging modality for shoulder and joint space infections, as demonstrated in this case, ultrasound is an appealing and less time-intensive method for quickly evaluating joint space fluid collection and tissue erosion. Not only can ultrasound guide arthrocentesis, but it also can expedite the involvement of the orthopedic surgery service from the ED, which is of the highest priority given the rate at which tissue deformity and joint space erosion can progress when infections such as this are left untreated. In this case, it is possible that, in lieu of waiting for several months to obtain an outpatient MRI, this patient could have been quickly evaluated and undergone a joint aspiration under POCUS guidance.

The ultrasonographic findings of osteomyelitis and septic arthritis are well described [5,6]. Ultrasound can be used to detect volumes as low as 1-2 ml of fluid in a joint capsule, which can guide a definitive diagnosis via aspiration, though synovial thickness, a typical finding, has not been proven to be particularly specific for septic arthritis [5]. Ultrasonographic findings, which can suggest progression to osteomyelitis, include cortical irregularities reflecting the underlying pathophysiology of periosteal elevation, such as those seen in this patient (Figures 1-4) [5]. These irregularities can be difficult to elucidate with ultrasound and require a skilled operator [6]. However, soft tissue fluid collections and subperiosteal abscess, which can be associated with osteomyelitis, can readily be appreciated using POCUS [6].

## Conclusions

Delays in diagnosis and treatment of septic arthritis can lead to a rapid destruction of the affected joint, causing significant morbidity and mortality. The patient in this case experienced severe delays in obtaining MRI imaging as an outpatient due to the COVID-19 pandemic. As patients are often unable to see their outpatient providers due to clinic closures and conversion to telemedicine, emergency physicians must be prepared to diagnose and treat these patients promptly, particularly in the setting of delays in obtaining definitive imaging.

It is imperative to remember that limitations of outpatient virtual and in-person medicine in critical cases continue to exist, and all providers must continue to have a high index of suspicion when patient conditions do not improve as predicted. This case demonstrates that, in lieu of waiting for months for an MRI and arthrocentesis, POCUS has the potential to be extremely helpful in the ED to rapidly diagnose and thus subsequently manage septic arthritis. Increasing the utilization of POCUS in both inpatient and outpatient settings to diagnose joint space infections has the potential to improve diagnostics and reduce wait times for treatment.
